# Supplementary material for: Optimality and sub-optimality in a bacterial growth law
Source: Nat Commun. 2017 Jan 19;8:14123. doi: 10.1038/ncomms14123 (PMC5253639; doi:10.1038/ncomms14123)
Supplement: Supplementary Information — Supplementary Figures, Supplementary Tables, Supplementary Notes, and Supplementary References [file ncomms14123-s1.pdf]

1. Supplementary Figures

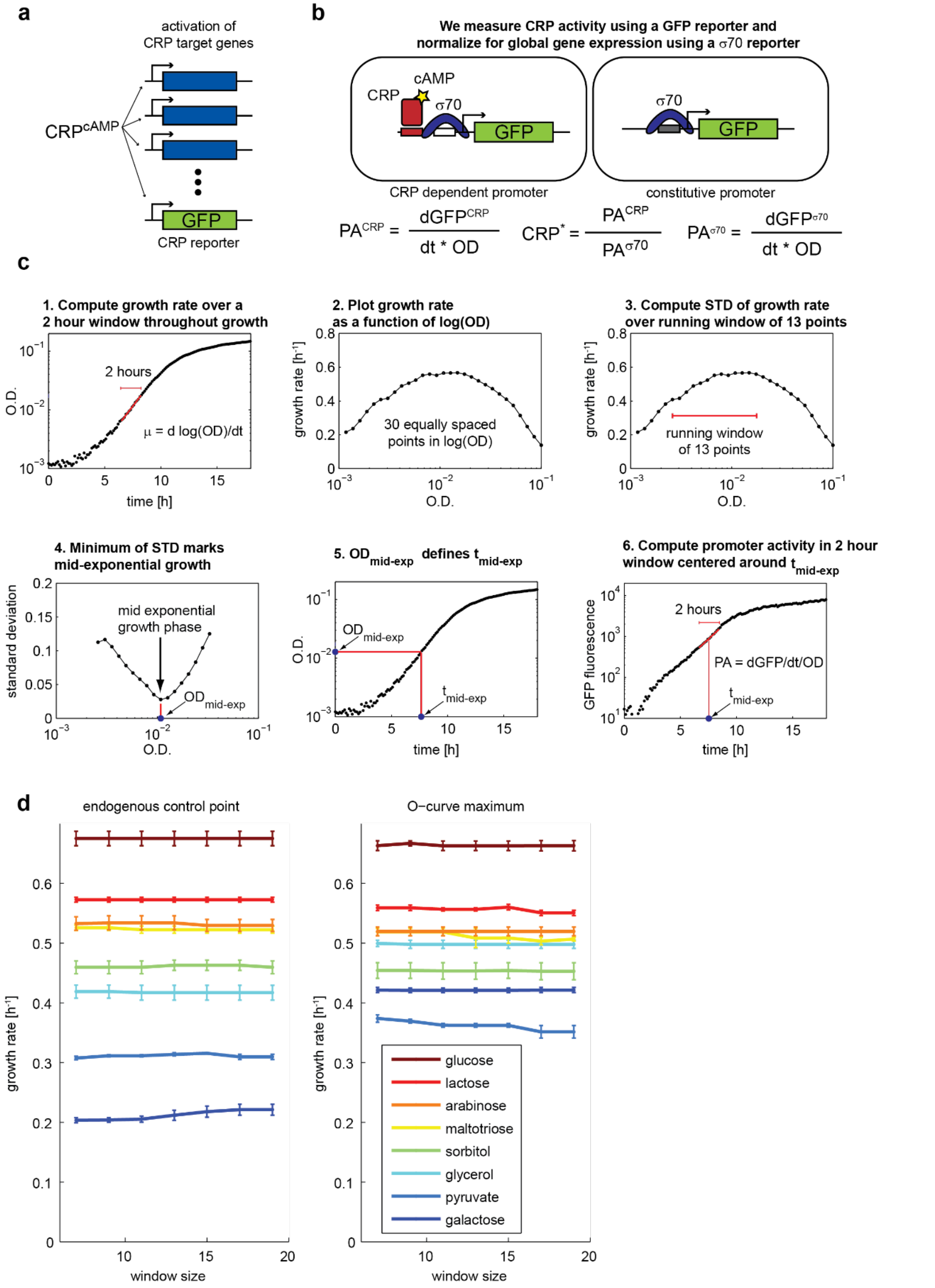

Supplementary Figure 1. Measurement of CRP activity using a GFP reporter. a. CRP-cAMP activates several hundred genes, including many genes involved in carbon catabolism and uptake.

To measure CRP activity we introduce a synthetic CRP-dependent GFP reporter on a low-copy plasmid [1]. b. We measure CRP activity as the ratio of the promoter activity of a CRP-dependent GFP reporter and an otherwise identical, but CRP-independent GFP reporter. This approach normalizes for global changes in gene expression associated with changes in growth rate and for condition dependent plasmid copy-number. The activities of the reporters were measured independently in two strains (whose only difference is the reporter plasmid they carry). Auto-fluorescence background was subtracted using a strain with a promoterless GFP. The three strains were grown in parallel under identical conditions. GFP and optical density (OD) were measured every 9 minutes in a robotic plate reader and promoter activity was calculated as the rate of GFP production per optical density (PA = dGFP/dt/OD). c. Method used to determine mid-exponential growth. Growth rate was computed by averaging over a 2 hour window (= 15 measurement points). To identify mid-exponential growth we plotted the growth rate as a function of log(OD) and identified the point with minimal standard-deviation of the growth rate within a running window of 13 points. The growth rate at this point was used for further analysis and the PA of CRP and σ70 reporters was calculated from a 2 hour window centered around the same time point. d. The growth rates at the endogenous control point and at the O-curve maximum were computed using the approach outlined in (c) using window sizes between 7 and 19 points for computing the standard deviation (step 4). The measured growth rates are robust to variation of the window size. Error bars are standard errors of the mean from 3 day-day repeats.

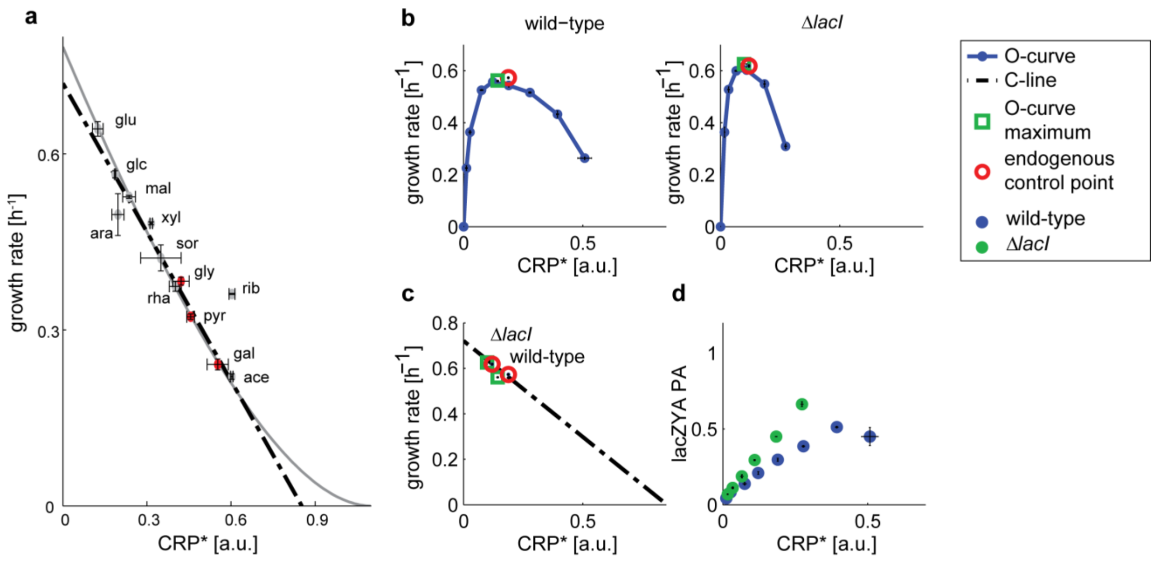

Supplementary Figure 2. CRP activity adapts optimally to increasing expression of lactose pumps by mutation of the lac repressor *lacI*. a. Data as in Figure 1c. Conditions where the endogenous growth rate differed by more than 5% from the O-curve maximum are labeled in red. glu: D-glucose, ara: L-arabinose, sor: D-sorbitol, gal: D-galactose, gly: glycerol, pyr: pyruvate, ace: acetate, rib: D-ribose, xyl: D-xylose, mal: maltotriose, glc: D-gluconate, rha: L-rhamnose. b. O-curves for growth on lactose in wild-type (data as Figure 1d), and *ΔlacI* mutant. CRP activity is nearly optimal in both genotypes (see Supplementary Table 1 for statistics). c. Observed and optimal CRP control points nearly coincide in both genotypes and are on the C-line. d. Promoter activity (PA) of the *lacZYA* promoter in both genotypes (normalized to the PA of the σ70 reporter). The PA of the *lacZYA* promoter is proportional to CRP activity in both genotypes, but the input function is steeper in a *ΔlacI* background, such that at the same CRP activity more *lacZYA* proteins are expressed in a *ΔlacI* mutant. Error bars are standard errors of the mean from 3 day-day repeats.

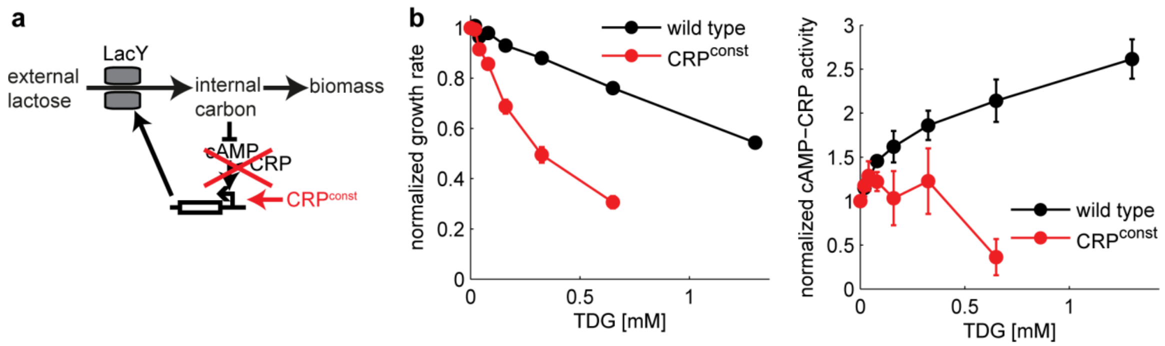

Supplementary Figure 3. Control of CRP activity makes *E. coli* less sensitive to inhibition of lactose uptake. a. To abolish control of CRP activity we mutated adenylate cyclase and CRP (*ΔcyaA Δcrp*). We supplemented this strain with a mutant version of CRP (CRP^{const}) that activates transcription independent of cAMP. CRP^{const} was expressed from a plasmid under control of the constitutive *bla* promoter [2]. b. Normalized growth rate and CRP activity of wild-type and CRP^{const} strains in lactose with increasing concentrations of the LacY inhibitor TDG. To correct for a slightly lower growth rate of the CRP^{const} strain in the absence of TDG the data is shown relative to the growth rate without TDG. Error bars are standard error of the mean from 5 day-day repeats. Where no error bars are visible the error was smaller than the size of the data point.

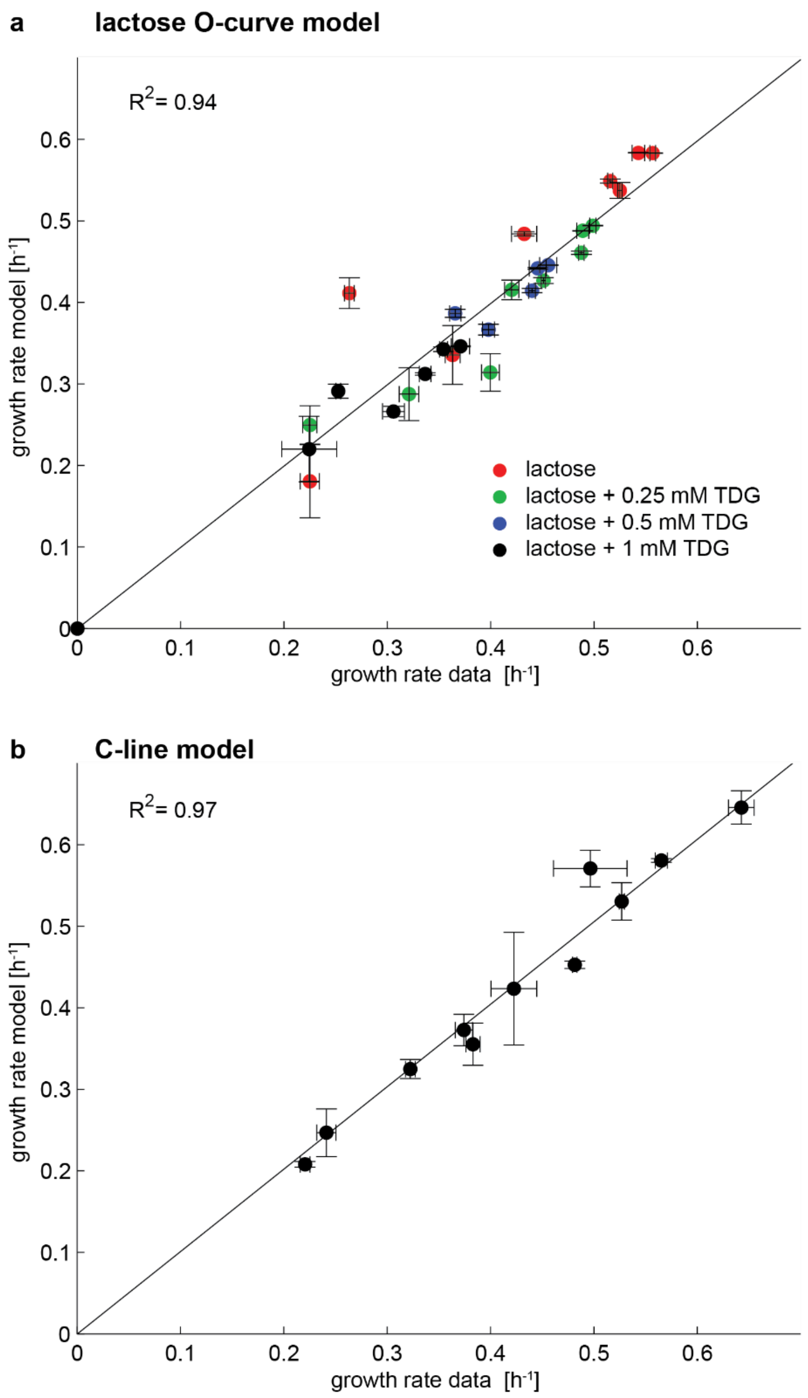

Supplementary Figure 4. Models of lactose O-curves and C-line fit the measured data well. a. Correlation of measured growth rates on lactose (+TDG) and the growth rates predicted by the O-curve model (see Figure 1g). Correlation coefficient R^2 = 0.94, p = 1.0e-19, root-mean-squared error RMSE = 0.04/h, explained variance EV = 94%. b. Correlation of measured growth rate and the growth rate predicted by the C-line model (grey line in Figure 1c). Correlation coefficient R^2 = 0.97, p = 6.9e-8, RMSE = 0.03/h, EV = 92%. Error bars in x are the standard error of the mean (s.e.m.) of growth rate measurements (3 day-day repeats). Error bars in y are the s.e.m. of the growth rate predicted by the model from 3 day-day repeats.

Model O-curves and C-line from best fit parameters

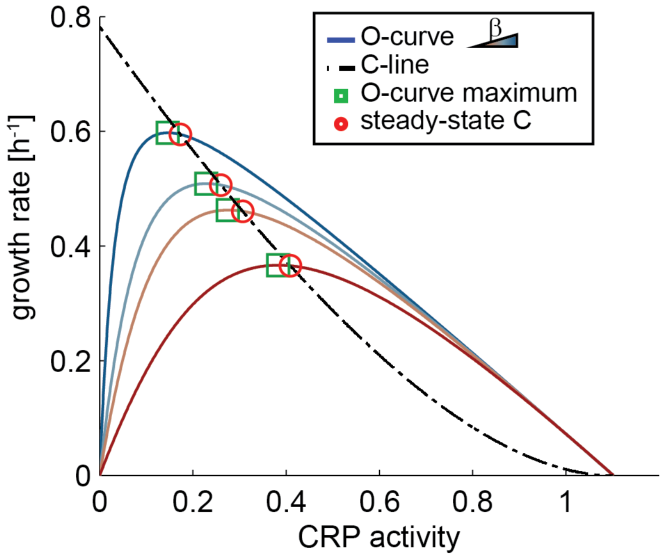

**best fit parameters**	TDG concentration			
	0 mM	0.25 mM	0.5 mM	1 mM
β [h^{-1}]	19.78	7.44	4.84	2.13

γ [h^{-1}]	0.78
k_1/k_2	1.88
k_f/k_2	1.71

**model**	TDG concentration			
	0 mM	0.25 mM	0.5 mM	1 mM
maximal growth rate [h^{-1}]	0.60	0.51	0.46	0.37
steady-state growth rate [h^{-1}]	0.59	0.51	0.46	0.37
error [h^{-1}]	0.003	0.003	0.002	0.001

**experiment**	TDG concentration			
	0 mM	0.25 mM	0.5 mM	1 mM
maximal growth rate [h^{-1}]	0.56	0.50	0.46	0.37
steady-state growth rate [h^{-1}]	0.57	0.50	0.44	0.36
error [h^{-1}]	-0.01	0.00	0.02	0.01

Supplementary Figure 5. Deviations from optimality of best fit model parameters are small and close to measured deviations from optimality. Model O-curves and C-line are plotted for parameters taken from fits to lactose + TDG experiments (Figure 1g). Deviation of O-curve maxima (green square) and observed CRP activity (red circle) is small and similar to experimentally observed deviations. Tables list best fit model parameters, and deviations from optimality for model and experiment.

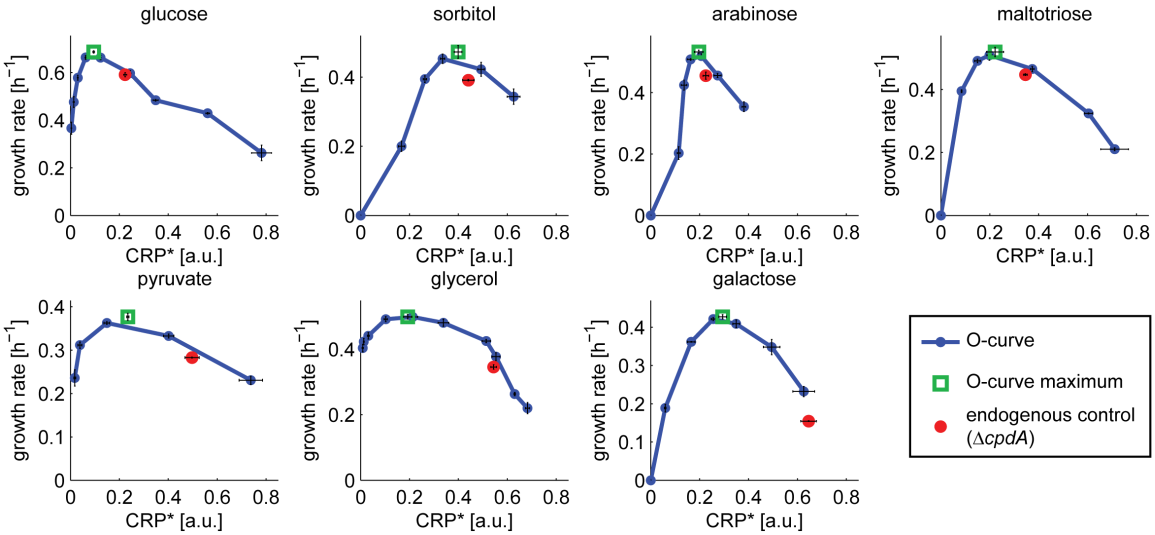

Supplementary Figure 6. Deletion of phosphodiesterase (*cpdA*) leads to suboptimal control of CRP activity. CRP activity and growth rate were measured in a *cpdA* deletion strain (full red circle) and is shown together with the measured O-curves from Figure 2. In the *cpdA* mutant CRP activity is sub-optimal under all conditions, as predicted by the model (p=0.01; 0.02; 0.04; 0.01; 0.001; 0.001; 0.0001 for the 7 conditions, one-sided t-test).

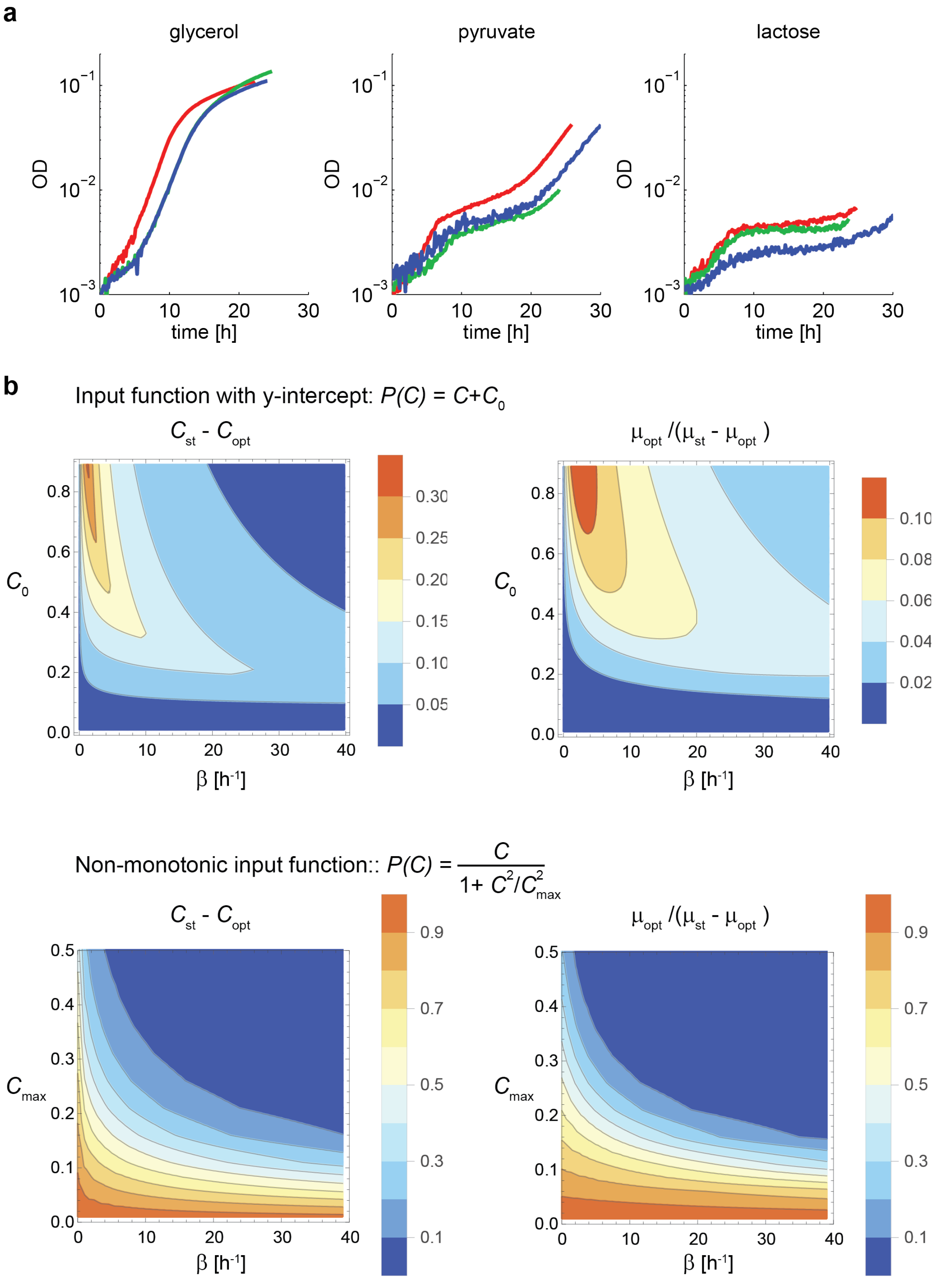

Supplementary Figure 7. cAMP is not required for growth on glycerol and pyruvate, leading to suboptimal CRP control. a. A *cyaA cpdA* deletion mutant was grown on the

indicated carbon sources without supplementation of cAMP to the growth medium. Shown is optical density (OD) over time for three day-day repeats (shown in red, green and blue). Growth at early time points is due to carry-over from pre-culture. On pyruvate, this initial growth is followed by a long lag phase (nearly 10 hours) followed by faster growth. No growth is detectable on lactose after the carry-over is consumed. This is consistent with tight CRP control of lactose genes. b. Dependence of predicted deviations from optimal growth rate on model parameters for non-proportional input functions. The relative error in growth rate and the error in CRP activity for input functions with a y-intercept (top) and for non-monotonic input functions (bottom) were numerically computed for a range of parameter values with $k1 = k2 = k_f$. Note that the error in growth rate decreases with increasing β. This explains why no deviation from the maximal growth rate is apparent in glucose, although the input functions of glucose transporters do have a y-intercept.

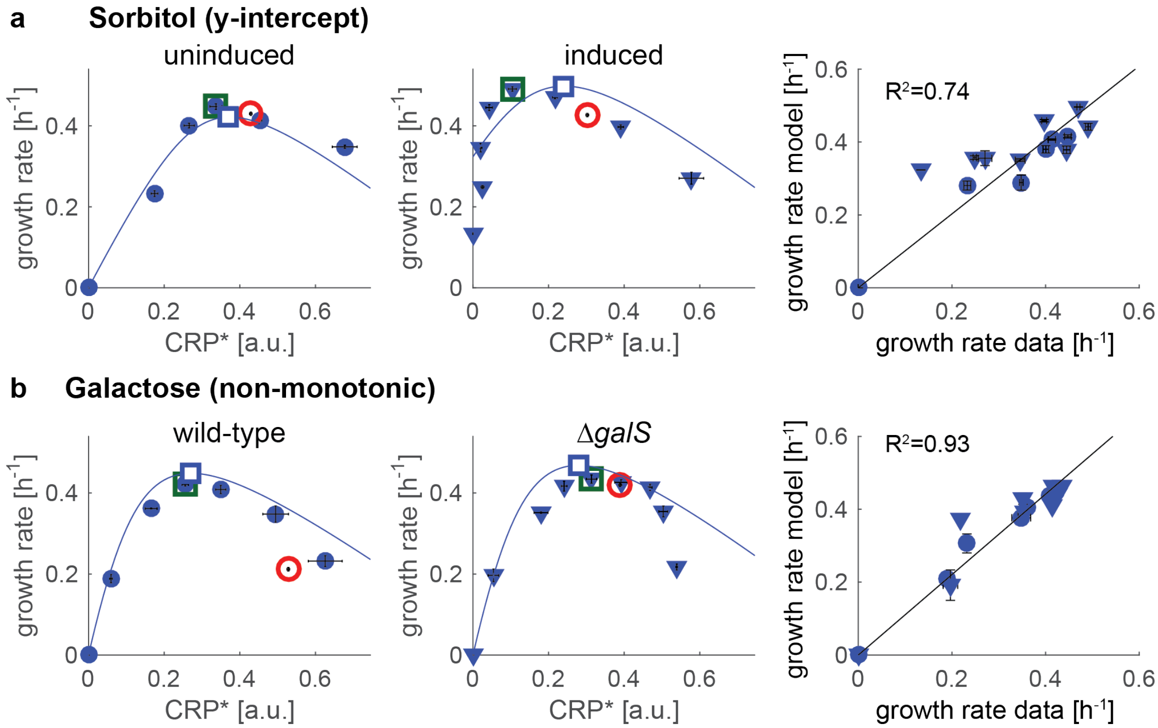

Supplementary Figure 8. Model quantitatively predicts the O-curve of the engineered systems. O-curve data for engineered and native sorbitol (a) and galactose (b) systems from Figure 4 are shown with the best fit models. The models for the native and engineered systems have equal values for all shared parameters (see Supplementary Note 3). Error bars are standard errors of the mean from 3 day-day repeats. Quality of fit for sorbitol: R^2 = 0.74, p-val = 8.1e-5, RMSE = 0.07/h, EV = 74%. Quality of fit for galactose: R^2 = 0.93, p-val = 1.4e-9, RMSE = 0.05/h, EV = 92%. Green square: measured O-curve maximum, blue square: predicted O-curve maximum, red circle: endogenous control point.

a

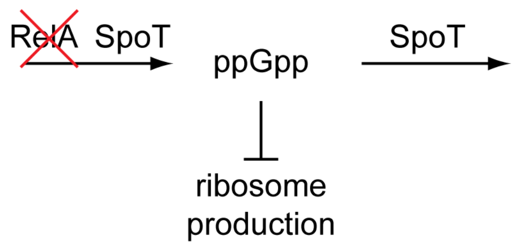

b

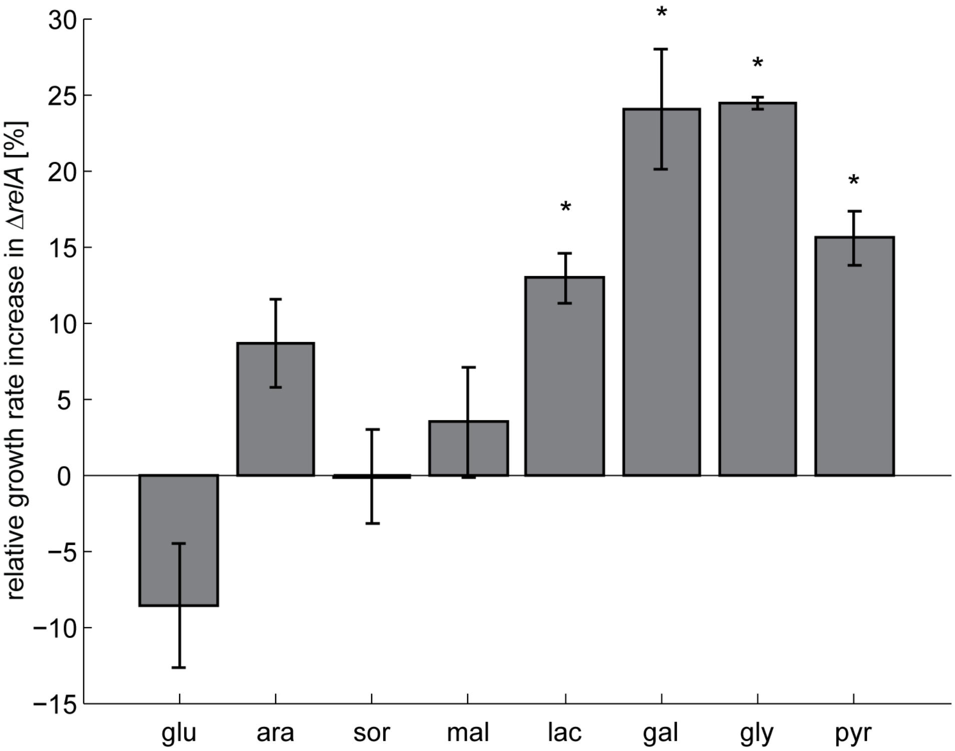

Supplementary Figure 9. Deletion of the ppGpp synthetase *relA* increases the growth rate on lactose, galactose, glycerol, and pyruvate. a. *E. coli* encodes two synthetases for ppGpp (a regulatory molecule that inhibits ribosome production): RelA and SpoT. SpoT is bifunctional and also degrades ppGpp. We deleted *relA* to reduce ppGpp production. *relA* is best known for its function in the stringent response, an acute inhibition of ribosome synthesis upon amino acid starvation (nutritional down-shift), but *relA* mutants also have mildly reduced basal levels of ppGpp during steady state growth [3]. b. Relative increase in growth rate of *ΔrelA* compared to wild-type. Growth rate significantly increased on lactose, galactose, glycerol, and pyruvate ($p = 0.008$; 0.012; 4.4e-5; 0.0078, respectively, marked with asterisk; $p > 0.05$ for other sugars, one-sided t-test). Exponential growth rate was computed from a linear fit to log(OD) during exponential growth between OD=0.003 and OD=0.03. Error bars are standard error of the mean from 3 day-day repeats.

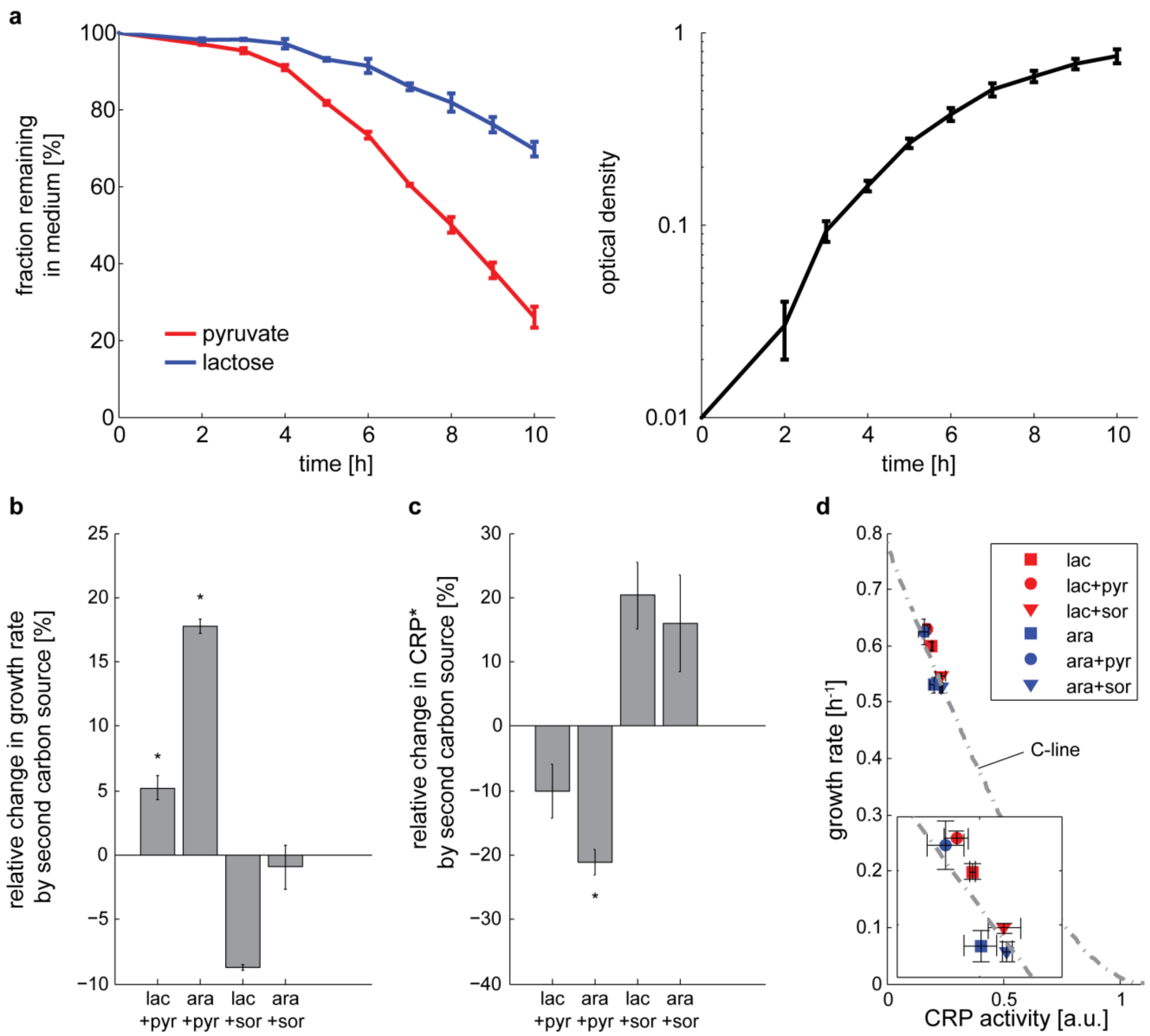

Supplementary Figure 10. Pyruvate is co-consumed with lactose and its addition improves growth rate on lactose and arabinose. a. Bacteria were grown in a medium containing a mixture of lactose (0.2%) and pyruvate (0.1%). The fraction of pyruvate and lactose remaining in the growth medium as a function of time, measured by HPLC, indicates co-consumption. Error bars are standard errors of the mean from 3 biologically independent cultures. b. Pyruvate, but not sorbitol, improves growth on lactose and arabinose. The growth rate of *E. coli* on lactose and arabinose (on which CRP activity is low) is improved by adding pyruvate to the growth medium (* $p<0.05$, one-sided t-test). Shown is the relative difference of the growth rate on lactose (or arabinose) alone and when pyruvate (or sorbitol) is added. A positive difference indicates improved growth rate upon addition of the second carbon source. No significant improvement, or a decrease in growth rate, was observed for addition of sorbitol. c. Relative change of CRP activity for same conditions as in b. d. The endogenous control point remains on the C-line in conditions with mixed carbon sources. The dotted line is the C-line model from Figure 1c. Error bars indicate standard error of the mean from 3 day-day repeats.

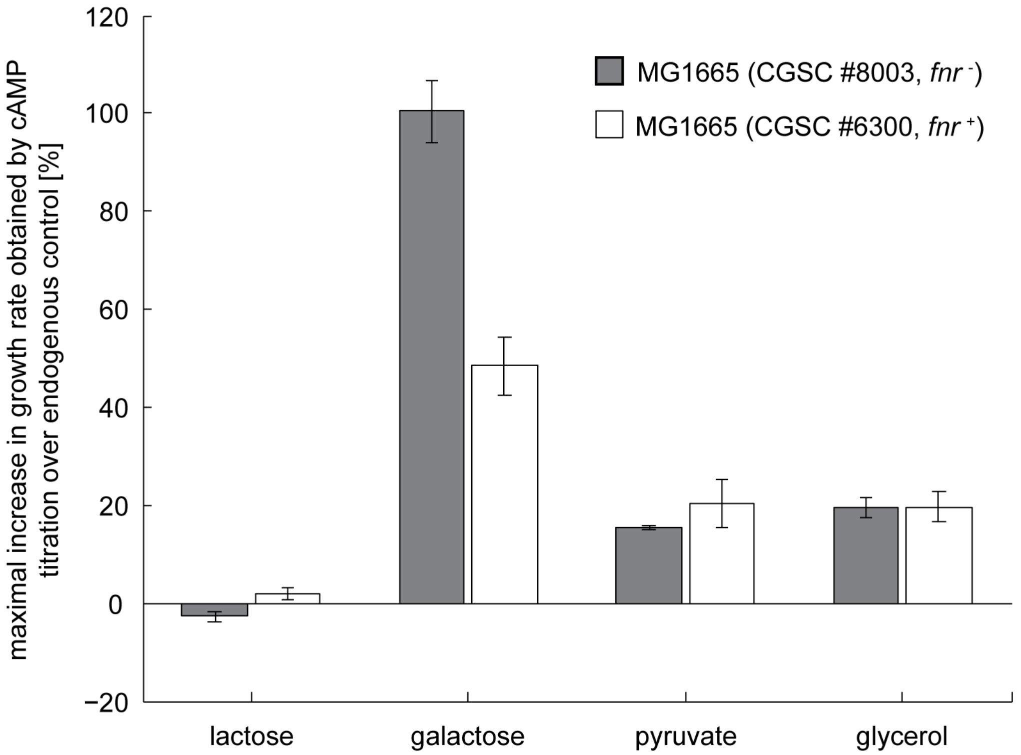

Supplementary Figure 11. Suboptimality on galactose, glycerol and pyruvate is not due to an *fnr* deletion. Shown is the relative growth rate difference of the endogenous control point and the maximum of the O-curve for two different MG1665 isolates, with and without a deletion of the genomic region around *fnr*. Both strains show a similar degree of sub-optimality for growth on the indicated carbon sources. Error bars are standard errors of the mean from 3 day-day repeats.

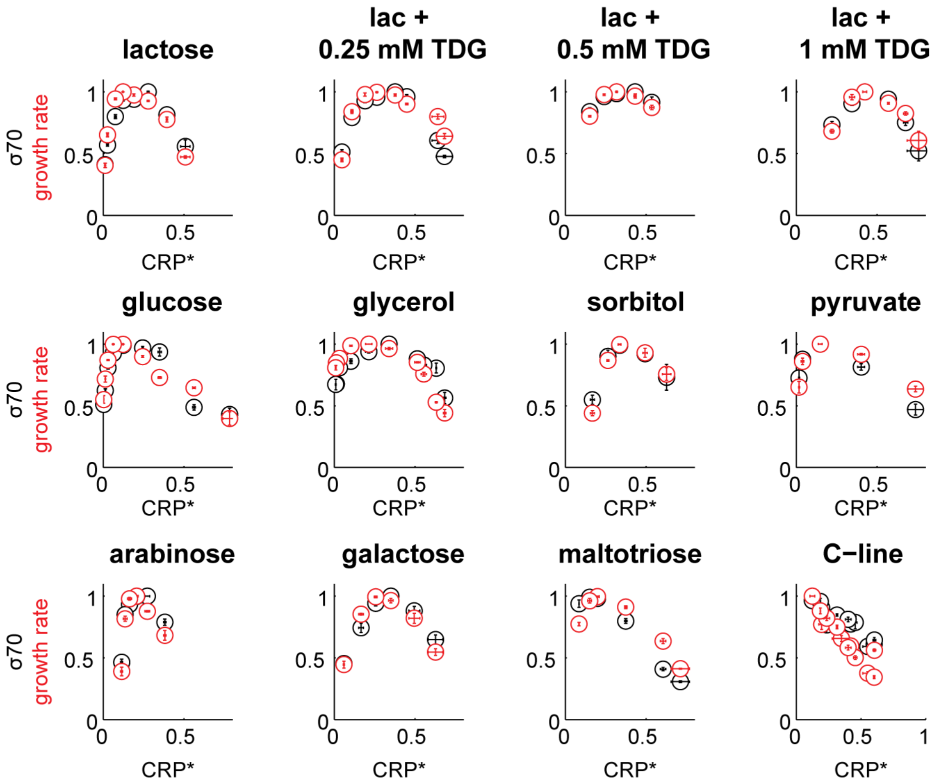

Supplementary Figure 12. The activity of the σ70 reporter scales with the growth rate. Promoter activity of the σ70 reporter (black) and growth rate (red) normalized to the maximum in each condition are shown as a function of CRP activity for O-curves on indicated carbon sources and for the C-line (same carbon sources as in Supplementary Figure 2a). Error bars are standard error of the mean from 3 day-day replicates.

2. Supplementary Tables

Supplementary Table 1. Relative difference of growth rate at O-curve maximum and at the endogenous control point

**condition**	**$(\mu_{max} - \mu_{end})/\mu_{end}$**	**Suboptimal (>5% difference)**	**p-value (one-sided t-test)**
lactose	-3%	no	0.94
lactose + 0.25mM TDG	-1%	no	0.82
lactose + 0.5mM TDG	4%	no	0.049
lactose + 1mM TDG	3%	no	0.15
lactose (*ΔlacI*)	-1%	no	0.2
glucose	-2%	no	0.82
sorbitol	-2%	no	0.81
arabinose	-3%	no	0.77
maltotriose	-2%	no	0.71
pyruvate	15%	yes	0.0005
glycerol	20%	yes	0.0027
galactose	101%	yes	0.0004
galactose (*ΔgalS*)	4%	no	0.028
sorbitol + *pZA31:srlAEBD* (uninduced)	4%	no	0.12
sorbitol + *pZA31:srlAEBD* (125ng/ml aTC)	15%	yes	0.02

Supplementary Table 2. Comparison of measured and predicted growth rates for the engineered sorbitol and galactose systems

	Measured maximal growth rate on O-curve ($\mu^{max}{}_{me}$) [h^{-1}]	Predicted maximal growth rate on O-curve§ ($\mu^{max}{}_{pr}$) [h^{-1}]	Growth rate at endogenous control point (μ_{end}) [h^{-1}]	Measured deviation from maximum ($\mu^{max}{}_{me}$ -μ_{end})/ μ_{end} [%]	Predicted deviation from maximum ($\mu^{max}{}_{pr}$ -μ_{end})/ μ_{end} [%]
Sorbitol *pTet* uninduced	0.45 +/- 0.01	0.42 +/- 0.01	0.43 +/- 0.004	4 +/- 2	3 +/- 2
Sorbitol *pTet* induced	0.49 +/-0.01	0.48 +/- 0.01	0.43 +/- 0.01	15 +/- 3	13 +/- 3
Galactose wild-type	0.42 +/- 0.004	0.39 +/- 0.004	0.21 +/- 0.01	100 +/- 6	86 +/- 9
Galactose Δ*galS*	0.41 +/- 0.01	0.44 +/- 0.01	0.42 +/- 0.01	4 +/- 1	-3 +/- 2

Data shown is the mean of 3 day-day repeats +/- s.e.m.

§ In order to calculate an error bar for the predicted O-curve maximum, we fitted the model to each of three day-day repeats and then took the mean and s.e.m. of these three fits.

Supplementary Table 3. Strains used in this study

Name	Genotype	description	reference
MG1655 (CGSC#: 8003)	*λ- rph-1 Δfnr-267*	all strains except MG1655 (CGSC#: 8003) and U486 are in this background	[5]
U472	*ΔcyaA ΔcpdA*		this study
MG1655z1	*pN25:tetR lacIq:lacI specR*	chromosomal integration of *tetR*	[6]
U473	*pN25:tetR lacIq:lacI specR ΔcyaA ΔcpdA*		this study
U477	*ΔlacI*		this study
U477	*ΔlacI ΔcyaA ΔcpdA*		this study
U480	*ΔcyaA Δcrp*		this study
U481	*ΔcpdA*		this study
U482	*ΔcyaA ΔcpdA ΔgalS*		this study
U483	*ΔgalS*		this study
U484	*ΔrelA*		this study
MG1655 (CGSC#: 8003)	*λ- rph-1*		[5]
U486	*λ- rph-1 ΔcyaA ΔcpdA*		this study

Supplementary Table 4.Plasmids used in this study

Name	description	reference
pU66	pSC101 origin, KanR, promoterless GFP reporter	[1]
pU66-HV2	as U66, but with CRP dependent promoter	[1]
pU66-HV13	as U66, but with constitutive σ70 promoter	[1]
pZA31:srlAEBD	*srlAEBD* genes under control of *Tet* promoter. Chloramphenicol resistance.	this study
pHA7*5	pBR322 backbone, AmpR, cAMP-independent CRP under control of *bla* promoter.	[2]

Supplementary Table 5. Primers used in this study

Name	sequence
oBT202	CCCAAGCTTATAAAACGAAAGGCTCAGTC
oBT203	CATGCCATGGTGAATTCGGTCAGTGCGT
oBT166	CCCAAGCTTGGGGCATTCTATAGTCTCACGG
oBT168	CATGCCATGGCATGCCTGAAGGAGAGAACAATGA

3. Supplementary Notes

Supplementary Note 1: Model description

We divide the proteome of the cell into three fractions: C, R and Q, following [7]. Proteome sector C stands for CRP regulated proteins, which include carbon catabolic enzymes and carbon transporters. R stands for all proteins involved in biomass production including ribosomes, translation and tRNA synthesis pathways, and amino acid biosynthesis genes. Sector Q includes all proteins which stay constant under the conditions studied here. Most proteins within one proteome sector maintain constant ratios throughout conditions [8,9], such that we can describe their relative abundance in a single variable per sector. We define the three sections as fractions of the total proteome to get

[1] $\tilde{Q} + \tilde{C} + \tilde{R} = 1$

If $\tilde{Q}$ is constant we can normalize C and R by a constant $(1 - \tilde{Q})$ to get:

[2] $C + R = 1, \text{with } C = \frac{\tilde{C}}{1-\tilde{Q}},\ R = \frac{\tilde{R}}{1-\tilde{Q}}$

where C and R are the fractions of the regulated proteome dedicated to carbon catabolism and biomass synthesis.

We condense all carbon catabolites to a single variable x, which represents the transition point between catabolism and anabolism (Figure 3a). We think of x as α-keto acids, which are known to inhibit cAMP synthesis [7] and carbon uptake [10], and are the precursors for amino acid synthesis. However, the exact molecular nature of x is not central for the purpose of this model.

The growth rate of cells is proportional to the rate of biomass synthesis by ribosomes R, which we model with Michaelis-Menten dependence on x with half-maximal point k_2 and maximal catalytic rate γ:

[3] $\mu = \gamma R \frac{x}{k_2 + x} = \gamma(1 - C)\frac{x}{k_2 + x}$

We model the partitioning between proteome allocation to C and R as a decreasing function of x: $f(\mathrm{x}) = \frac{k_\mathrm{f}}{k_\mathrm{f} + x}$, given the well-characterized phenomenon of carbon catabolite repression, in which internal carbon represses the production of cAMP and thereby inhibits allocation to the C-Sector [7,11]. The function $f(x)$ ranges between 0 and 1, where f=1 means allocation exclusively to carbon catabolism and f=0 means allocation exclusively to biomass synthesis. The parameter k_f determines the half-maximal point of $f(x)$.

Assuming Michaelis-Menten kinetics for the turnover of carbon into biomass we can describe the dynamics of the concentration of x as follows:

[4] $\dot{x} = p\left(\beta P(C)\frac{k_1}{k_1+x} - \gamma R\frac{x}{k_2+x}\right)$

We can neglect dilution of x through growth, because x is used up for biomass synthesis much more quickly than it is diluted.

The carbon import rate is $\beta P(C)\frac{k_1}{k_1+x}$, where β is the maximal import rate (which depends on external sugar availability and quality), and $\frac{k_1}{k_1+x}$ describes a reduction of net carbon import rate when internal carbon levels are high. This reduction can be due to allosteric inhibition of the transporters by x [10] or due to other processes, such as carbon efflux.

The proportionality factor p accounts for the number of units of x needed to produce one unit of biomass (C or R). $P(C)$ describes the regulation of the limiting enzyme for carbon uptake and catabolism by CRP. If carbon transport is a limiting step, $P(C)$ corresponds to the input function of the carbon transporter gene. For many carbon sources, carbon pump expression is nearly proportional to C [8,13], such that $P(C) = C$.

C sector dynamics are described by:

[5] $\dot{C} = \mu\left(\frac{k_\mathrm{f}}{k_\mathrm{f}+x} - C\right)$

The production rate of C is the total protein production rate μ, multiplied by the control function $f(x) = \frac{k_\mathrm{f}}{k_\mathrm{f}+x}$ which sets the allocation of resources into carbon catabolic activity C. The dilution of C is also dictated by the exponential growth rate and is given by μC, so that at steady state $C=f(x)$, as described in the main text.

To reduce the number of parameters, we define unit-less variables:

[6] $\tilde{x} = \frac{x}{k_2}, a = \frac{k_1}{k_2}, \widetilde{k_\mathrm{f}} = \frac{k_\mathrm{f}}{k_2}, \tilde{p} = \frac{p}{k_2}$

such that the full model is described by:

[7] $\frac{d\tilde{x}}{dt} = \tilde{p}(\beta P(C)\frac{a}{\tilde{x}+a} - \gamma(1-C)\frac{\tilde{x}}{\tilde{x}+1})$

[8] $\frac{dC}{dt} = \mu\left(\frac{\widetilde{k_\mathrm{f}}}{\tilde{x}+\widetilde{k_\mathrm{f}}} - C\right)$

[9] $\mu = \gamma(1-C)\frac{\tilde{x}}{\tilde{x}+1}$

Supplementary Note 2: Computation of O-curves

To measure the O-curves we uncoupled CRP regulation from internal carbon concentrations and set CRP activity using external cAMP (i.e. eq. [8] is ignored and C is set to a constant). Eq. [7] allows computation of $\tilde{x}(C)$ at steady-state ($\dot{\tilde{x}} = 0$) which we use to substitute $\tilde{x}$ in eq. [9] to get:

[10] $\mu(C) = \frac{a\gamma - aC\gamma + a\beta P(C) + \sqrt{a(4(1-C)\beta\gamma P(C) + a((C-1)\gamma + \beta P(C))^2)}}{2(a-1)}$

Substitution of *P(C)* leads the following O-curve models for different input functions:

Proportional input function (*P(C)=C*):

[11] $\mu_{\text{proportional}}(C) = \frac{a(C(\beta-\gamma)+\gamma) - \sqrt{a(4(1-C)C\beta\gamma + a(\gamma - C(\beta+\gamma))^2)}}{2(a-1)}$

Input function with a y-intercept (*P(C)* = *C*+C_0):

[12] $\mu_{\text{y-int}}(C) = \frac{a((C+C_0)\beta + \gamma - C\gamma) - \sqrt{a(4(1-C)(C+C_0)\beta\gamma + a((C+C_0)\beta - (1-C)\gamma)^2)}}{2(a-1)}$

Non-monotonic input function ($P(C) = \frac{C}{1 + (C/C_{\max})^2}$):

[13] $\mu_{\text{non-mon}}(C) =$

$$\frac{-a\,C\,C_{\max}{}^2\beta - a(1-C)(C^2 + C_{\max}{}^2)\gamma + \sqrt{a(4(1-C)CC_{\max}{}^2(C^2+C_{\max}{}^2)\beta\gamma + a(CC_{\max}{}^2\beta - (1-C)(C^2+C_{\max}{}^2)\gamma)^2)}}{2(a-1)(C^2+C_{\max}{}^2)}$$

Supplementary Note 3:Fitting O-curve model to data

To fit the model to the O-curve data (Figure 1c, Supplementary Figure 8), we used equations [11]-[13] with the modification

$$C = \frac{C^{\text{measured}}}{C_{\text{m}}}$$

to account for the arbitrary units of maximal experimental CRP*. The parameters that we fit are:

1. γ, maximal rate of *R*-sector proteins, assumed to be constant for all experiments across all carbon sources.
2. β, maximal carbon import rate (carbon source specific)
3. C_{m}, CRP* where the complete proteome is allocated to carbon catabolism and the growth rate=0, constant for all experiments across all carbon sources.
4. $a = \frac{k_1}{k_2}$, constant for experiments in same carbon source, but variable across carbon sources.
5. C_0, $C_{\max}$, parameters of non-monotonic input functions

To avoid over-fitting we determined the parameters C_{m} and γ from fits to the O-curves in glucose, sorbitol and maltotriose (Figure 2a). On these three carbon sources the right tail of the O-curve converged to nearly the same linear decline. This behavior is expected in the absence of cost of excessive enzyme activity, a prerequisite to get good estimates of γ and C_{m}.

To calculate the fits, we minimized the mean squared error between model and measurements using the NMinimize function of Mathematica. As an additional constraint, we demanded C_m to be bigger than the highest measured CRP activity.

For fits to lactose O-curves (Figure 1g) we fitted the parameters α and β in eq. [11], demanding that α was constant for experiments with different concentrations of the LacY inhibitor TDG.

For fits of model O-curves in Supplementary Figure 8 we fitted the parameters α, β, C_0 (for the sorbitol system), and $C_{\max}$ (for the galactose system) using eq. [11] for the proportional system and eq. [12] (sorbitol) and eq. [13] (galactose) for the non-proportional system. We demanded the same values for the shared parameters α and β for the engineered and non-engineered systems, leaving C_0 (for sorbitol) and $C_{\max}$ (for galactose) as the only additional parameter for the engineered system. In order to calculate an error bar for the predicted O-curve maximum in Supplementary Table 2, we fitted the model to each of three day-day repeats and then took the mean and s.e.m. of these three fits.

Supplementary Note 4: Computation of the C-line

At steady state $C_{st} = \frac{\widetilde{k_\mathrm{f}}}{\tilde{x}+\widetilde{k_\mathrm{f}}}$ (eq. [8]). We can inverse this relation to get

[14] $$\widetilde{x_\mathrm{st}} = \widetilde{k_\mathrm{f}}\left(\frac{1}{C_\mathrm{st}} - 1\right)$$

and substitute $\tilde{x}$ in eq. [9] to obtain the growth rate as a function of the carbon sector C:

[15] $$\mu_{\mathrm{C-line}} = \frac{\gamma(1-C)^2\widetilde{k_\mathrm{f}}}{C+\widetilde{k_\mathrm{f}}(1-C)}$$

The C-line crosses 0 when C = 1, and is equal to γ when C = 0.

Supplementary Note 5: Fitting C-line model to data

Using the γ from the O-curve fits to the O-curves of experiments in glucose, sorbitol and maltotriose (see Supplementary Note 3) we determined k_f by fitting eq. [15] to the growth rate and CRP activity measured for growth of the wild-type in 11 different carbon sources (Figure 1c). We excluded ribose, which was an outlier from the C-line. In the presence of ribose, several genes involved in nucleic acid synthesis are expressed at lower levels [14]. In the model this leads to a reduction of sector Q, and hence a proportional increase in C and R. In our reduced model, where we normalize out Q, this translates into increased γ and β. In agreement with this model, we find that the O-curve has a steeper right tail on ribose (data not shown). The slope and y-intercept of the C-line scale with γ, such that ribose is expected to fall on a different C-line. It can

therefore not be used to fit k_{f}. To calchulate the fits, we minimized the mean square error between model and measurements using the NMinimize function of Mathematica.

4. Supplementary References

1. Kaplan, S., Bren, A., Zaslaver, A., Dekel, E. & Alon, U. Diverse two-dimensional input functions control bacterial sugar genes. *Mol. Cell* **29,** 786–92 (2008).
2. Aiba, H., Nakamura, T., Mitani, H. & Mori, H. Mutations that alter the allosteric nature of cAMP receptor protein of Escherichia coli. *EMBO J.* **4,** 3329–32 (1985).
3. Atherly, A. G. Escherichia coli mutant containing a large deletion from relA to argA. *J. Bacteriol.* **138,** 530–534 (1979).
4. Gerosa, L., Kochanowski, K., Heinemann, M. & Sauer, U. Dissecting specific and global transcriptional regulation of bacterial gene expression. *Mol. Syst. Biol.* **9,** 658 (2013).
5. Soupene, E. *et al.* Physiological Studies of Escherichia coli Strain MG1655: Growth Defects and Apparent Cross-Regulation of Gene Expression. (2003). doi:10.1128/JB.185.18.5611
6. Hart, Y. *et al.* Robust control of nitrogen assimilation by a bifunctional enzyme in E. coli. *Mol. Cell* **41,** 117–27 (2011).
7. You, C. *et al.* Coordination of bacterial proteome with metabolism by cyclic AMP signalling. *Nature* 1–6 (2013). doi:10.1038/nature12446
8. Keren, L. *et al.* Promoters maintain their relative activity levels under different growth conditions. *Mol. Syst. Biol.* **9,** 701 (2013).
9. Hui, S. *et al.* Quantitative proteomic analysis reveals a simple strategy of global resource allocation in bacteria. *Mol. Syst. Biol.* **11,** 784 (2015).
10. Doucette, C. D., Schwab, D. J., Wingreen, N. S. & Rabinowitz, J. D. α-Ketoglutarate coordinates carbon and nitrogen utilization via enzyme I inhibition. *Nat. Chem. Biol.* **7,** 894–901 (2011).
11. Magasanik, B. Catabolite Repression. *Cold Spring Harb. Symp. Quant. Biol.* **26,** 249–256 (1961).
12. Cayley, S. & Record, M. T. Roles of cytoplasmic osmolytes, water, and crowding in the response of Escherichia coli to osmotic stress: biophysical basis of osmoprotection by glycine betaine. *Biochemistry* **42,** 12596–12609 (2003).
13. Aidelberg, G. *et al.* Hierarchy of non-glucose sugars in Escherichia coli. *BMC Syst. Biol.* **8,** 133 (2014).
14. Shimada, T., Kori, A. & Ishihama, A. Involvement of the ribose operon repressor RbsR in regulation of purine nucleotide synthesis in Escherichia coli. *FEMS Microbiol. Lett.* **344,** 159–65 (2013).
